# Associations of bullying victimization with problematic internet gaming and problematic social media use among adolescents: moderators and differences

**DOI:** 10.1186/s13034-025-01008-x

**Published:** 2025-12-14

**Authors:** Franziska Neumayer, Vanessa Jantzer, Alina Killer, Stefan Lerch, Michael Kaess

**Affiliations:** 1 Department of Child and Adolescent Psychiatry, Center for Psychosocial Medicine, University Hospital Heidelberg, Blumenstr. 8, 69115 Heidelberg, Germany; 2 Faculty of Behavioral and Cultural Studies, Institute of Psychology, Heidelberg University, Hauptstr. 47-51, 69117 Heidelberg, Germany; 3 University Hospital of Child and Adolescent Psychiatry and Psychotherapy, University of Bern, Bolligenstrasse 111, Stöckli, 3000 Bern 60, Switzerland

**Keywords:** Problematic internet gaming, Problematic social media use, Bullying, Adolescents

## Abstract

**Background:**

Bullying, problematic internet gaming, and problematic social media use are concerning phenomena, especially among youth. However, studies including all three of them are scarce. Therefore, this study investigated the associations between bullying victimization and the two internet-related outcomes. Furthermore, differences between problematic internet gaming and problematic social media use regarding the individual and moderating effects of gender, age, educational background and mental health problems were examined.

**Methods:**

Adolescents (*N* = 6,735; 48.85% females) answered a school-based survey on bullying, problematic internet gaming, problematic social media use and mental health problems. The age ranged from grade 5 with *M* = 10.77 years (*SD* = 0.68) to grade 9 with *M* = 14.75 years (*SD* = 0.87) and overall *M* = 12.73 years (*SD* = 1.60). A-level school students represented higher educational background (39.52%) while B-level school students represented lower educational background (60.48%). Multilevel modelling was used to examine the associations of bullying victimization with problematic internet gaming and problematic social media use as well as the influences of gender, school grade as a correlate of age, school type and mental health problems.

**Results:**

Victims of bullying showed higher odds for problematic internet gaming and problematic social media use. Overall, boys showed higher levels of problematic internet gaming, whereas girls showed higher levels of problematic social media use. Younger adolescents reported higher odds for problematic internet gaming, while no age effect was found for problematic social media use. Students with lower educational background and those with more mental health problems reported more problematic social media use than problematic internet gaming. Mental health problems moderated the association of bullying victimization with problematic internet gaming and problematic social media use, with stronger relations for students with less mental health problems. Furthermore, gender was a significant moderator for problematic social media use but not for problematic internet gaming, with a stronger association for boys.

**Conclusions:**

Bullying victimization is strongly related to different types of problematic internet use. As differences in the impact on problematic internet gaming and problematic social media use can be identified, prevention should also consider gender, age, educational background and mental health problems.

*Trial registration* DRKS00028183.

**Supplementary Information:**

The online version contains supplementary material available at 10.1186/s13034-025-01008-x.

## Introduction

Bullying is a common and concerning phenomenon among youth. It is characterized by aggressive behavior that involves a power imbalance between the victim and the perpetrator(s) and is usually repeated multiple times [1]. Traditional forms of bullying such as direct (e.g., physical, verbal aggressions) or indirect bullying (e.g., relational aggressions) typically occur at school or in neighborhoods, while the relatively new phenomenon of cyberbullying is carried out through electronic devices (e.g., verbal, relational online-aggressions) [1, 2]. A recent meta-analysis found that 24.32% of adolescents reported being a victim of traditional bullying and 11.10% experienced cyberbullying [3]. Most studies report a large overlap of bullying in the two contexts, i.e., the majority of victims who are cyberbullied also experience traditional bullying [3, 4]. These prevalence estimates are particularly alarming, as all forms of bullying victimization can lead to various short- and long-term psychological problems, including depression, self-harm and suicidality, as well as physical problems like headaches or back pain [3, 5–11]. Thus, bullying in any form constitutes a global health concern for youths which is particularly important considering that mental and physical well-being are internationally recognized public health priorities [12, 13]. In this study, the term bullying victimization comprises experiences of direct, indirect, as well as cyberaggression, unless stated otherwise.

### Bullying victimization, problematic internet gaming and problematic social media use

A fairly new mental health issue among adolescents that has been found to be associated with bullying victimization are internet use disorders [14–16]. These internet-related addictions have risen for the past decades and have become an increasing concern in society, clinical practice and research [17]. Of all internet activities, gaming and social media use appear to be most popular among youth [18]. Therefore, investigating problematic internet gaming (PIG) and problematic social media use (PSMU) in adolescents is of particular relevance. Highlighting this, Internet Gaming Disorder (IGD) as a pathological extent of PIG, has been included in the fifth edition of the Diagnostic and Statistical Manual of Mental Disorders (DSM-5; [19]), and Gaming Disorder was recognized in the latest revision of the International Classification of Diseases (ICD-11; [20]). Both manuals describe a recurrent problematic use of (online or offline) games during the last 12 months. According to the DSM-5, at least five of nine criteria must be fulfilled to diagnose IGD: (1) excessive preoccupation with gaming, (2) withdrawal symptoms if gaming is not possible, (3) development of tolerance, (4) loss of control over gaming, (5) loss of interest in other activities, (6) continuation of excessive gaming despite knowing about psychosocial consequences, (7) deceiving others about the scope of gaming, (8) use of gaming to escape negative moods and (9) jeopardizing or losing relationships or education opportunities because of the gaming. In contrast to gaming, PSMU is not part of the diagnostic manuals and still lacks consensus regarding its definition. Generally, PSMU is characterized by addiction criteria similar to those defining IGD but concerning social networking sites, messengers or blogs [21–23]. With prevalence rates of 8.80% for IGD and 5.00% for PSMU, both problematic behaviors constitute a mental health concern among adolescents [15, 21, 24].

### Comparing PIG and PSMU

Some studies compared PIG and PSMU regarding various parameters such as comorbid symptoms, psychological distress and metacognitions to better understand similarities and differences between these two common internet-related addictions [25–29]. Similar underlying mechanisms have also been posited by a popular theoretical model – the Interaction of Person-Affect-Cognition-Execution (I-PACE) model [30]. This model suggests that addictive behaviors such as PIG or PSMU emerge from the interaction between predisposing core characteristics of a person, the affective and cognitive responses to certain triggers, as well as executive functions. Considering the relationships of PIG and PSMU with bullying, victimization might be one potential trigger of these addictive behaviors [14–16]. Potential predisposing core characteristics could be gender, age, educational background and mental health problems. These characteristics might be similar or different between PIG and PSMU across adolescents in general or specifically with respect to bullying victims.

#### The role of gender in PIG and PSMU

Regarding gender, a variety of meta-analyses suggest that IGD is generally more prevalent in boys [15, 31, 32], while girls show a higher prevalence for PSMU [21, 33]. In line with these findings, male bullying victims seem to be more at risk for the development of PIG [34, 35], whereas for female victims, the relationship between social media use and bullying victimization was found to be stronger [14, 16]. One explanation for these gender-specific patterns could be differences in their coping styles: While boys were found to prefer avoidant coping (e.g., by escaping into a virtual gaming world), girls appeared to seek social support (e.g., in an online community) [36]. Despite these findings, some research points out that PIG is also common among girls, for instance, depending on the gaming genre, and that games are becoming increasingly attractive to females [37, 38]. Thus, PIG might still be a hidden problem in girls. In addition, some studies on PSMU suggest that females are often over-represented, which might influence the findings [21, 24].

#### The role of age in PIG and PSMU

Results on the role of age are even more controversial. For instance, in the age group of 12- to 18-year-olds, some studies reported a higher probability of PIG for younger participants [39], whereas other studies found higher probabilities for older age [40]. In contrast, a meta-analysis with studies on 9- to 21-year-olds reported no association between mean age and the prevalence of IGD [41]. For PSMU, the findings are also inconsistent. While one study found higher prevalence rates for 13- and 15-year-olds compared to 11-year-olds [42], another study reported the highest rate at 13 years followed by 11 years and lastly by 15 years [43]. In addition, findings on how bullying victimization interacts with age regarding internet use disorders in adolescents are inconsistent [16, 44].

#### The role of educational background in PIG and PSMU

In some countries there are different types of secondary schools to which students transition after primary school – among other factors – based on their academic achievements. Typically, these school types consist of high schools preparing students for university enrollment, and alternatively, vocational high schools. Thus, the former school type is usually regarded as higher educational background. Some studies suggest that vocational high school students might be more prone to problematic internet use, perhaps because of less awareness for its negative consequences [45]. However, while PIG seems to be associated to a lower education [46–48], the role of the educational background in terms of PSMU [42, 49] and the interaction with bullying victimization regarding PIG and PSMU is still inconclusive [35].

#### The role of mental health problems in PIG and PSMU

One further potential predisposing variable for the development of problematic internet use suggested by the I-PACE model is psychopathology [30]. Indeed, research indicates that internalizing mental health problems such as symptoms of depression or anxiety, and externalizing problems like attention deficit hyperactivity disorder, are very common in internet-related addictions [50, 51] and that mental health problems are associated with PIG [52] and PSMU [53]. Furthermore, research suggests that mental health problems mediate the associations between bullying victimization and PIG [54] as well as PSMU [55]. However, to our knowledge, no study has investigated the influence of mental health problems on the relationship between victimization and PIG in comparison to PSMU.

### The current study

Generally, studies comparing PIG and PSMU in the context of bullying victimization and further influential variables are still scarce [56, 57]. However, this would contribute conceptionally to a better understanding of the components suggested by the I-PACE model regarding different behavioral addictions [30]. In addition, from a clinical perspective, identifying potential groups at risk of PIG or PSMU may contribute to the development of targeted prevention and intervention strategies. Thus, the current study takes a first step to fill the research gaps identified above by examining a large-scale, cross-sectional sample of adolescents, which was conducted as part of the evaluation of a new, school-based anti-bullying program [58]. We aim to better understand the associations between bullying victimization and PIG, as well as PSMU, and to identify potential differences between them regarding the individual as well as the moderating effects of gender, age, educational background and mental health problems. Specifically, due to the inconsistent findings of prior studies, we hypothesized

1. that bullying victimization would be significantly associated with PIG and PSMU to a similar extent,
2. that there would be no differences between PIG and PSMU in terms of the association with gender, age, educational background and mental health problems and
3. that there would be no differences in the moderating effects of gender, age, educational background and mental health problems between bullying victimization and PIG compared to PSMU.

## Methods

### Procedure

The data used in the current study originates from the evaluation of the anti-bullying program *Bullying&You* (German: *Mobbing&Du*; [58]). The evaluation was conducted by the Department of Child and Adolescent Psychiatry at the University Hospital Heidelberg, and was funded by the Baden-Wuerttemberg Foundation (*Baden-Wuerttemberg Stiftung*). Primary and secondary schools in the German state of Baden-Wuerttemberg were informed about the option to participate in the program and to enroll in the study (for further details see: [58]). Overall, 13 primary and 27 secondary schools were recruited and randomly assigned to an intervention group or a waiting control group. From 2022 to 2023, students in grade 3 and 4 (primary school) and 5 to 9 (secondary school) completed the first of three annual online surveys including questions on bullying and mental health. Students in grades 5 to 9 additionally filled in questionnaires on problematic internet use. These baseline data were collected prior to the implementation of the anti-bullying program. For organizational reasons, the annual surveys were administered either at the end of the school year in June/July (10 schools), at the beginning of the school year in October/November (9 schools), or at half-year change in March/April (8 schools). Data collection took place at school during class hours (maximum 90 min). Teachers read standardized instructions aloud before the students started to fill out the self-administered survey.

The current study used the dataset to conduct secondary analyses independent of the evaluation study and focused only on the baseline data collected in the 27 secondary schools (of the intervention as well as the control group).

### Participants

Of a total of *N* = 8,951 eligible students, *N* = 6,884 (76.91%) provided consent to participate in the study. Due to incomplete datasets, *N* = 149 participants (2.16%) were deleted, resulting in *N* = 6,735 students who were included in the analysis. As 2.16% of missing data is well below the threshold of common practice [59, 60], data were analyzed using the complete case sample. However, it should be noted that data were not completely missing at random. Students in grade 7 and 8 had fewer missing data than those in grade 5, 6 and 9 (χ^2^ (4) = 29.00, *p* < .001) and students from A-level schools (= *Gymnasium*: high school with academic focus, grades 5 to 12 or 13, required to enroll at university) showed fewer missing data compared to students from B-level schools (= *Realschule/Werkrealschule/Gemeinschaftsschule*: more practical high school education, grades 5 to 9 or 10, allows for vocational training, but is insufficient to enroll at university) (*OR* = 3.00, 95% confidence interval [CI]: [1.98;4.54], *p* < .001).

For the complete case sample, the average age across grades 5 to 9 was 12.73 years (*SD* = 1.60). The age ranged of from grade 5 with *M* = 10.77 years (*SD* = 0.68) to grade 9 with *M* = 14.75 years (*SD* = 0.87). Overall, 143 classes belonged to A-level schools representing higher educational background, while 272 classes belonged to B-level schools representing lower educational background. Of the 6,735 participants, 17.64% (*n* = 1,188) were classified as victims of bullying. Further sample characteristics comparing participants depending on their bullying status with the full sample are displayed in Table 1.

**Table 1:** Sample characteristics based on either the full sample (*N* = 6,735), bullying victims (*n* = 1,188) or non-victims (*n* = 5,547)

	Full sample	Bullying victim	No bullying victim
	*n* (%)	*n* (%)	*n* (%)
Gender			
Male gender	3,445 (51.15%)	565 (47.56%)	2,880 (51.92%)
Female gender	3,290 (48.85%)	623 (52.44%)	2,667 (48.08%)
Grade			
Grade 5	1,316 (19.54%)	249 (20.96%)	1,067 (19.24%)
Grade 6	1,427 (21.19%)	240 (20.20%)	1,187 (21.40%)
Grade 7	1,417 (21.04%)	270 (22.73%)	1,147 (20.68%)
Grade 8	1,309 (19.44%)	228 (19.19%)	1,081 (19.50%)
Grade 9	1,266 (18.80%)	201 (16.92%)	1,065 (19.20%)
School type			
A-level school	2,662 (39.52%)	401 (33.75%)	2,261 (40.76%)
B-level school	4,073 (60.48%)	787 (66.25%)	3,286 (59.24%)
IGD	951 (14.12%)	329 (27.69%)	622 (11.21%)
SMD	787 (11.69%)	295 (24.83%)	492 (8.87%)

	*M (SD)*	*M (SD)*	*M (SD)*
Internet Gaming Disorder Scale	2.01 (2.15)	2.99 (2.56)	1.80 (1.99)
Social Media Disorder Scale	1.66 (2.13)	2.73 (2.64)	1.43 (1.93)
SDQ (without bullying item)	13.48 (6.07)	17.78 (6.19)	12.56 (5.63)

Calculation of percentages (%) in cells: numerator = *n* in cell, denominator = *n* of column; IGD = Internet Gaming Disorder; SDQ = Strengths and Difficulties Questionnaire; SMD = Social Media Disorder

### Measures

#### Bullying victimization

Bullying was assessed using an adapted version of the Bullying Screening since it is a short instrument which still comprises different facets of bullying [61, 62]. In previous adolescent samples the victimization questions had a Cronbach’s alpha of 0.73, and predictive validity was demonstrated [62, 63]. In the current sample the Cronbach’s alpha was 0.67. The relatively low internal consistency observed is likely attributable to the scale’s primary function as a screening tool for different forms of bullying experiences. Although these forms share similarities, they represent distinct, yet related, constructs. More precisely, the Bullying Screening contains six questions: three on bullying victimization (direct, indirect, cyber) and three on perpetration (direct, indirect, cyber). Each question includes a clear definition and concrete examples of bullying to ensure validity. In this study, only the questions on victimization were analyzed (supplementary material, Table A1). A six-point scale was used to rate the frequency within the past six months. If participants indicated “often (2–3 times a month)” or more on at least one of the three questions on bullying victimization, they were classified as victims. This cut-off is widely used in research [64].

#### Problematic internet gaming

The German version of the Internet Gaming Disorder Scale was used to assess PIG [65]. In previous adolescent samples the reliability coefficients were between 0.56 and 0.83, and criterion validity was demonstrated [66, 67]. In the current sample the Cronbach’s alpha was 0.76, and Harman’s single factor test showed 35.09% of explained variance (see supplementary material). The scale was introduced by the sentence: “The following questions are about your use of computer games in the last year. By computer games, we mean all games played on a computer, tablet, game console or smartphone (offline and online).“ It includes nine questions with one question per DSM-5 criterion for IGD [19]: preoccupation (“Have there been periods when all you could think of was the moment that you could play a game?”), tolerance (“Have you felt unsatisfied because you wanted to play more?”), withdrawal (“Have you been feeling miserable when you were unable to play a game?”), persistence (“Were you unable to reduce your time playing games, after others had repeatedly told you to play less?”), escape from negative feelings (“Have you played games so that you would not have to think about annoying things?”), continuation despite problems (“Have you had arguments with others about the consequences of your gaming behavior?”), deception (“Have you hidden the time you spend on games from others?”), displacement (“Have you lost interest in hobbies or other activities because gaming is all you wanted to do?”) and conflicts (“Have you experienced serious conflicts with family, friends or a partner because of gaming?”). The dichotomous response format (“yes”=1, “no”=0) leads to a sum score ranging between 0 and 9. This sum score with a higher value indicating a greater severity of PIG in the last 12 months was used in the current study. In line with the DSM-5, participants with a sum score of five or more were classified as having IGD in Table 1 [19].

#### Problematic social media use

PSMU was assessed by the German version of the Social Media Disorder Scale [27]. In previous adolescent samples the reliability coefficients were between 0.57 and 0.81, and good convergent and criterion validity were demonstrated [23, 27]. In the current sample the Cronbach’s alpha was 0.80, and Harman’s single factor test showed 39.85% of explained variance (see supplementary material). The scale was introduced by the sentence: “The following questions relate to your use of social media such as WhatsApp, SnapChat, social networking sites such as Facebook, Twitter, Instagram, Google+, Pinterest and forums and blogs/weblogs.” Similar to the DSM-5 criteria for IGD [19], it examines the same nine symptoms with similar wording as the Internet Gaming Disorder Scale [65] but in relation to the use of social media (e.g., for preoccupation: “Have you regularly found that you can’t think of anything else but the moment that you will be able to use social media again?”). Analogously, the dichotomous response format (“yes”=1, “no”=0) leads to a sum score ranging between 0 and 9. This sum score with a higher value indicating a greater severity of PSMU in the last 12 months was used in the current study. A sum score of at least five was classified as indicative of Social Media Disorder (SMD) in Table 1.

#### Mental health problems

Mental health problems were assessed by the Strengths and Difficulties Questionnaire (SDQ; [68]) which is a behavioral screening questionnaire and is not intended for diagnostic purposes. Its five scales consist of five questions each, resulting in overall 25 questions. The five scales are emotional symptoms (e.g., “I am often unhappy, depressed or tearful.”), conduct problems (e.g., “I fight a lot. I can make other people do what I want.”), hyperactivity/inattention (e.g., “I am restless, I cannot stay still for long.”), peer relationship problems (e.g., “I get along better with adults than with people my own age.”) and prosocial behavior (e.g., “I am helpful if someone is hurt, upset or feeling ill.”). A three-point scale was used to rate the degree of agreement (“not true”=0, “somewhat true”=1, “certainly true”=2) concerning the last six months. To calculate a total difficulties score for the main analyses, the first four scales were added, resulting in a range of 0–40. In previous adolescent samples the Cronbach’s alpha of the total difficulties score was = .80, and validity was demonstrated [69]. One of the questions on the peer relationship problems scale addresses bullying (“Other children or young people pick on me or bully me”), which might cause confounding. Therefore, this question was excluded, resulting in a maximum score of 38. To ensure the usual maximum range of 0–40, the total difficulties score was divided by 38 and then multiplied by 40. Neglecting one question in the total difficulties score did not affect the internal consistency in the current sample: Cronbach’s alpha including the bullying question was .80, Cronbach’s alpha excluding the bullying question was .79. Moreover, the factor structure remained coherent as principal axis factoring for the total difficulties score showed a change in loadings of maximum 6.50%.

For further analyses, the scores of the emotional symptoms and peer relationship problems scales were summed to obtain an internalizing score, and the scores of the conduct problems and hyperactivity/inattention scales were summed to an externalizing score. In previous adolescent samples the Cronbach’s alpha of the internalizing score was 0.66, the Cronbach’s alpha of the externalizing score was 0.76, and validity was demonstrated [70]. In the current sample the Cronbach’s alpha of the externalizing score was 0.70. When the bullying question was excluded, the maximum range of the internalizing score was 18 instead of 20. To ensure the usual maximum range, the internalizing score was divided by 18 and then multiplied by 20. This did not affect the internal consistency in the current sample: Cronbach’s alpha including the bullying question was 0.77, Cronbach’s alpha excluding the bullying question was 0.75. In addition, the factor structure remained coherent (principal axis factoring: maximum change in loadings = 3.29%).

### Statistical analysis

#### Modeling approach

Multilevel modelling was used to examine the associations of the independent variables bullying victimization (yes, no), gender (boy, girl), grade (grade 5, 6, 7, 8, 9; as a correlate of age), school type (A-level, B-level) and mental health problems (total difficulties score: 0–40) with PIG and PSMU. The model is graphically illustrated in Figure A1 in the supplementary material. We operationalized PIG and PSMU using their nine binary items. The associations were modelled as direct effects from the independent variables to the PIG and PSMU questions using a logit link function. Effects and thresholds were constrained to be equal across the nine questions but may differ between PIG and PSMU. This specification accounts for the bounded count nature of the data, similar to a binomial regression with nine trials, while avoiding violations of the Gaussian residual assumption. Simultaneously, it remains close to a continuous sum score in which all items are weighted equally, as prescribed in the manual for diagnosis. The constraints were validated using a principal component factor analysis showing one dominating factor on which all items loaded approximately equally (see Harman’s single factor test in the supplementary material).

#### Hierarchical structure

The data had a three-level structure: participants nested within classes and classes nested within schools. The null model of a binomial regression with nine trials showed a small intraclass correlation coefficient (ICC) on the school-level (PIG: ICC = 0.01, 95% CI [0.00;0.01]; PSMU: ICC = 0.01, 95% CI [0.00;0.02]). The school-level predictor school type mostly explained the variance. A likelihood ratio tests indicated no significant improvement from adding random effects at the school level (PIG: χ²(1) = 0.26, *p* = .61; PSMU: χ²(1) = 3.33, *p* = .07). Given the minimal unexplained school-level variance, random effects at the school level were omitted. Instead, two random intercepts were specified at the class level (one for PIG and one for PSMU), with unconditioned covariance.

#### Estimation and interpretation

Data processing and descriptive statistics were processed using Stata 18. The model was estimated using the “TWOLEVEL” analysis in Mplus 8.10 with the robust maximum likelihood estimator (MLR). Results are reported as odds ratio (OR) referring to odds of fulfilling a PIG or PSMU criterion. A change of more than one criterion is interpreted as large effect and a change of less than 0.25 criterion is interpreted as small effect. This translates to a large (*OR* ≤ 0.64, *OR* ≥ 1.56), medium (0.64 < *OR* ≤ 0.90, 1.12 ≤ *OR* < 1.56) and small (0.90 < *OR* < 1.12) effect size. For continuous variables with standard deviation (SD), the thresholds were transformed by raising them to the power of 1/SD. Wald tests were used to assess potential differences in effects between PIG and PSMU and comparison to the null model. Statistical significance was set to α = 0.05. Estimates and covariance matrices from Mplus were imported into Stata and results were plotted using the marginal command, assuming a two-level binomial regression with nine trials.

#### Further analyses

We increased the granularity of bullying victimization and calculated the model by replacing the dichotomous predictor bullying by three dichotomous variables direct, indirect and cyberbullying. In the same manner, we split mental health problems into internalizing and externalizing problems.

Interactions of bullying victimization with gender, grade, school type and mental health problems were examined in separate models. In each model, an additional interaction term was added as an independent variable acting separately on the PIG and PSMU questions. The models were compared to models without interaction terms.

### Ethics

The study was conducted in accordance with the Declaration of Helsinki, was approved by the Ethics Committee of the Medical Faculty of the University of Heidelberg (S-471/2020) and the Ministry of Education of the state of Baden-Wuerttemberg and registered in the World Health Organization trial registry (*Deutsches Register Klinischer Studien*; DRKS00028183). Leaflets with information about the study were handed out to all students and their caregivers. In addition, a member of the research team informed the students about the study in class. The caregivers were invited to contact the researchers in case of questions, and had the option to refuse the participation of their child (i.e., opt-out). All students included in the study provided active electronic informed consent at the beginning of the online survey by answering the question “Would you like to participate in the study?” with checking the box “Yes, I would like to participate in the study” (as opposed to “No, I do not want to or my parents do not want me to participate in the study”). If students did not consent, their data was only used for an annual school-specific report as part of the prevention program.

Participant privacy was protected by using randomly assigned login IDs (random combination of digits and letters) to register on the start page of the survey. If students gave consent to participate in the study, they were asked to create an individual code. This code allows linking the answers in the annual surveys to the same student without compromising participant confidentiality. Since only cross-sectional data were used in the current study, no linking by individual codes was necessary.

## Results

### Fixed effects of bullying victimization, gender, grade, school type and mental health problems

The model including bullying victimization, gender, grade, school type and mental health problems showed a good model fit (χ^2^ (16) = 3033.89, *p* < .001).

Bullying victimization was significantly associated with PIG (*OR* = 1.23, 95% CI [1.13;1.34], *p* < .001, medium effect) and PSMU (*OR* = 1.26, 95% CI [1.13;1.40], *p* < .001, medium effect), showing a higher risk for PIG and PSMU for victims than non-victims. These associations did not differ between PIG and PSMU (*OR* = 1.02, 95% CI [0.93;1.13], *p* = .64). For further analyses, two additional models with three forms of bullying victimization (direct, indirect, cyber) instead of overall bullying victimization were conducted. Model 1 assumed that all three forms of bullying have the same effect on PIG or PSMU, respectively, and model 2 assumed that all three forms of bullying have different effects on PIG or PSMU, respectively. Since model 2 showed no better model fit than model 1 (χ2 (4) = 5.75, *p* = .22), no analyses by form of bullying are reported below. However, results on the three bullying forms are included in the supplementary material (Table A2).

For gender, boys had a higher risk for PIG (*OR* = 2.25, 95% CI [2.08;2.42], *p* < .001, large effect), while girls had a higher risk for PSMU (*OR* = 0.79, 95% CI [0.72;0.86], *p* < .001, medium effect). These gender differences between PIG and PSMU were significant (*OR* = 0.35, 95% CI [0.32;0.38], *p* < .001, large effect).

Regarding grade, a significant effect was found for PIG (χ^2^ (4) = 55.52, *p* < .001) with a tendency of lower risk for PIG at older age (grade 5 versus 6: *OR* = 0.88, 95% CI [0.79;0.99], *p* = .03, medium effect; grade 6 versus 7: *OR* = 0.96, 95% CI [0.86;1.07], *p* = .49; grade 7 versus 8: *OR* = 0.87, 95% CI [0.77;0.98], *p* = .02, medium effect; grade 8 versus 9: *OR* = 0.88, 95% CI [0.78;1.00], *p* = .04, medium effect). In contrast, no significant effect was found in terms of PSMU (χ^2^ (4) = 6.95, *p* = .14). The effect of age differed significantly between PIG and PSMU (χ^2^ (4) = 93.58, *p* < .001).

For school type, B-level students had a higher risk for PIG (*OR* = 1.12, 95% CI [1.04;1.21], *p* < .01, medium effect) and PSMU (*OR* = 1.21, 95% CI [1.11;1.32], *p* < .001, medium effect) than A-level students. Significant differences were found between PIG and PSMU with a stronger influence of school type on PSMU than PIG (*OR* = 1.08, 95% CI [1.00;1.17], *p* < .05, small effect).

Regarding mental health problems, the more difficulties reported, the higher was the risk of PIG (*OR* = 1.11, 95% CI [1.10;1.12], *p* < .001, large effect) and PSMU (*OR* = 1.13, 95% CI [1.12;1.14], *p* < .001, large effect). The associations differed significantly between PIG and PSMU with a stronger influence of mental health problems on PSMU than PIG (*OR* = 1.02, 95% CI [1.01;1.02], *p* < .001, small effect). For further analyses, additional models with internalizing and externalizing problems instead of overall mental health problems were conducted. Model 1 assumed that both forms of mental health problems have the same effect on PIG or PSMU, respectively, and model 2 assumed that both forms have different effects on PIG or PSMU, respectively. Model 2 showed a better fit than model 1 (χ2 (2) = 33.11, *p* < .001). Since the main results of the model with internalizing and externalizing problems were the same as those of the model with overall mental health problems, the findings of the further analyses are included in the supplementary material (Table A3).

### Interactions of bullying victimization with gender, grade, school type and mental health problems

**Fig. 1:**
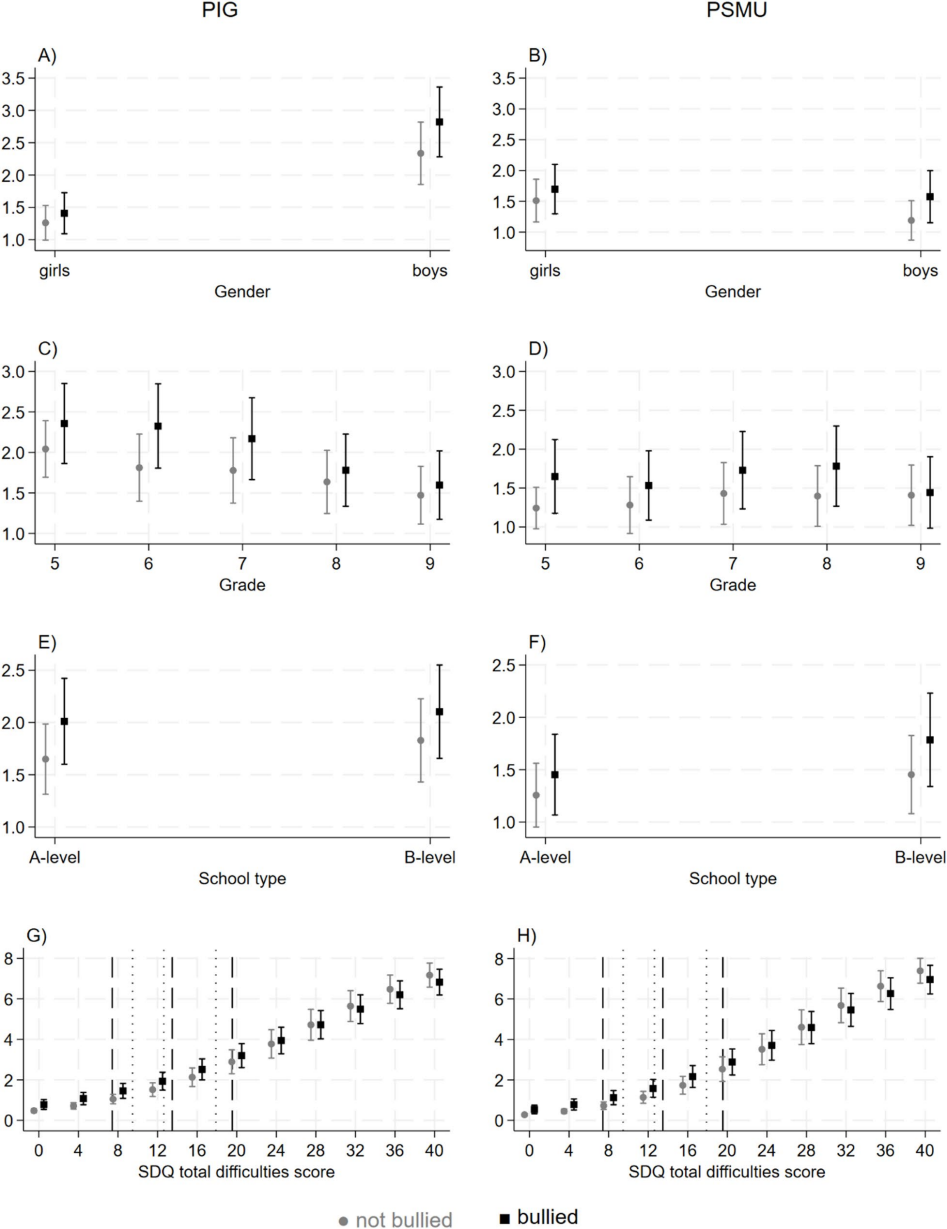
Interaction effects on problematic internet gaming and problematic social media use between bullying victimization and gender, grade, school type and mental health problems. *Note*: Illustrated are marginal predicted means and 95% confidence intervals; PIG: A, C, E, G; PSMU: B, D, F, H; gender: **A**, **B**; grade: **C**, **D**; school type: **E**, **F**; mental health problems: **G**, **H**; dashed vertical lines: mean +/- 1 standard deviation, dotted line: 25th percentile, median, 75th percentile; PIG = problematic internet gaming; PSMU = problematic social media use; SDQ = Strengths and Difficulties Questionnaire

In the paragraphs below, results of the interaction terms of the respective interaction models are presented, while a complete representation of all parameters can be found in the supplementary material (Table A4). Furthermore, all interactions are displayed in Fig. 1.

The model including the interaction of bullying victimization with gender showed a trend toward a better fit compared to the model without the interaction (χ^2^ (2) = 4.93, *p* = .09). The interaction effect between bullying and gender was not significant for PIG (*OR* = 1.15, 95% CI [0.98;1.35], *p* = .10), but for PSMU (*OR* = 1.21, 95% CI [1.01;1.45], *p* = .04, medium effect) with a stronger association between bullying and PSMU for boys than for girls. There were no significant differences in these interaction effects between PIG and PSMU (*OR* = 1.06, 95% CI [0.89;1.26], *p* = .53).

Regarding grade, the model including the interaction with bullying victimization did not demonstrate a better fit compared to the model without the interaction (χ^2^ (8) = 10.60, *p* = .23). No significant interaction was found with bullying neither for PIG (χ^2^ (4) = 4.71, *p* = .32) nor for PSMU (χ^2^ (4) = 3.70, *p* = .45), and these effects did not differ between PIG and PSMU (χ^2^ (4) = 6.70, *p* = .15).

Similarly, the model including the interaction of bullying victimization and school type did not fit better than the model without the interaction (χ^2^ (2) = 2.60, *p* = .27). Furthermore, the interaction effect for bullying and school type was not significant neither for PIG (*OR* = 0.93, 95% CI [0.79;1.10], *p* = .39) nor for PSMU (*OR* = 1.09, 95% CI [0.89;1.33], *p* = .42) and there were no significant differences between PIG and PSMU (*OR* = 1.17, 95% CI [0.96;1.41], *p* = .11).

For mental health problems, the model including the interaction with bullying victimization showed a significantly better fit compared to the model without the interaction (χ^2^ (2) = 10.95, *p* < .01). A significant interaction effect was found for PIG (*OR* = 0.98, 95% CI [0.97;0.99], *p* = .01, medium effect) and for PSMU (*OR* = 0.98, 95% CI [0.96;0.99], *p* < .01, medium effect) with stronger associations between mental health problems and PIG or PSMU, respectively, for non-victims than victims. The interaction effects did not differ between PIG and PSMU (*OR* = 0.99, 95% CI [0.98;1.01], *p* = .35). For further analyses, additional models with internalizing or externalizing problems instead of overall mental health problems were conducted. The model on the interaction of bullying victimization and internalizing problems revealed a significantly better fit compared to the model without the interaction (χ^2^ (2) = 12.54, *p* < .01). The main results were the same as for the model regarding overall mental health problems (supplementary material, Table A5 and Figure A2). For the model on the interaction of bullying victimization and externalizing problems, no better fit was found in comparison to the model without the interaction (χ^2^ (2) = 2.73, *p* = .26; supplementary material, Table A6 and Figure A3).

## Discussion

The current study investigated the associations between bullying victimization and PIG, as well as PSMU, and potential differences in the individual and moderating effects of gender, age, educational background and mental health problems between the two internet-related outcomes.

### Association of bullying victimization with PIG and PSMU

In line with previous research, our study found that bullying victimization was significantly associated with PIG [15] as well as PSMU with a medium effect size [14, 16, 71]. As hypothesized, there was no significant difference in the two outcomes. Therefore, victims of bullying showed higher odds for both problematic behaviors alike, which adds new insights to the few existing studies evaluating both in the same sample [56]. One possible explanation for this finding, as suggested by the I-PACE model, could be that victims of bullying try to cope with their negative feelings triggered through their experiences by escaping into an online-world [30]. The I-PACE model further suggests that a lack of inhibitory control may fuel this maladaptive emotion regulation strategy which was supported by a recent meta-analysis [30, 72]. As the use of gaming or social media to relieve negative moods is also one of the diagnostic criteria, this might not be surprising [19, 23]. In addition, this escape strategy was described as one of the mechanisms which contributes to the maintenance of internet addiction through negative reinforcement [73, 74]. Furthermore, victims of bullying might use the internet to compensate their lack of social belonging via digital contacts as suggested by the social compensation hypothesis [74, 75]. However, since cross-sectional data were analyzed, it also has to be considered that problematic internet use may not be the consequence but rather the cause of bullying experiences. For instance, adolescents who excessively game or use social media might be more likely to be bullied due to their deviation from the peer norm [76]. Additionally, online games or social media platforms can be the locations where cyberbullying occurs, thus, excessive use could increase the risk of becoming a victim [77–79].

### Individual and moderating effects of gender

Contradicting our hypothesis, boys generally reported higher levels of PIG while girls reported higher levels of PSMU with large or medium effect sizes, respectively. These findings add to prior studies suggesting that gender effects constitute a main difference between PIG and PSMU [15, 21, 33]. Thus, gender might be considered as a potential core characteristic in the I-PACE model [30]. One explanation for this difference may be that the competitive character of internet games is more attractive to boys, whereas the interpersonal focus of social media is more attractive to girls [33].

Regarding the moderating effects, no effect was found for gender on the association of victimization with PIG, which deviates from previous findings [34, 35]. Interestingly, and in contrast with previous findings, victimization moderately increased the odds more for boys than for girls to report PSMU [14, 16]. One explanation could be that victimized boys try to find support and connection via social media [75]. Alternatively, boys who excessively spend their time on the “girls domain” social media might be more likely to become a target of (cyber-)bullying [80, 81]. This means, we assume that these boys might be bullied by other boys because they deviate from the gender-stereotypical male norm; likewise, these boys might be bullied by girls on social media platforms because they “invaded” their female domain. However, these surprising results should be interpreted with caution, since characteristics of our sample might have contributed to them (see sample selection in section “Strengths, limitations and future directions”). What is noteworthy – since the comparison between PIG and PSMU was not significant (as hypothesized) – gender may not be regarded as a major differentiating factor in terms of bullying victimization.

### Individual and moderating effects of age

Another significant difference between PIG and PSMU, contradicting our hypothesis, was found concerning age: while no significant age effect was found for PSMU, younger adolescents were more likely to engage in PIG with mostly moderate effect sizes. This finding adds further insights to the ambiguous research regarding the relationship between age and PIG, and might prompt the consideration of age as a potential core characteristic in the I-PACE model in terms of PIG [30, 39]. In our view, one explanation for this finding could be that some characteristics of games (e.g., regularly taking care of one’s avatar, reaching certain milestones within a particular time frame to receive rewards) may render games especially addictive for younger adolescents due to their lower levels of inhibitory control [82].

No moderating effects of age were found on the association of bullying victimization with PIG and PSMU and, thus – as hypothesized – also no differences between them. As younger age groups are also already severely affected by bullying and internet-related addictions, this underscores the need for prevention concerning bullying as well as PIG/PSMU already at an early age.

### Individual and moderating effects of educational background

Aligning with existing research regarding differences in the educational background, students with lower educational background (B-level versus A-level schools) showed greater odds for PIG with a medium effect size [46–48] and – contradicting our hypothesis – we found this association to be even stronger for PSMU. Thus, educational background might be further investigated as a potential core characteristic in the I-PACE model [30]. However, due to the cross-sectional design, it is unclear whether lower education contributes to problematic internet use, for instance, due to less awareness of negative consequences, or whether excessive internet use leads to a neglect of school and homework and, thus, lower academic achievement [45, 83]. We assume that for B-level students, social media platforms may be even more attractive than gaming, because (in contrast to some games) they usually do not require complex decision making or cognitive skills [84]. However, due to the small effect size found for the difference between PIG and PSMU, further investigation is recommended.

Since no moderating effects of educational background were found on the association of bullying victimization with PIG and PSMU and – as hypothesized – also no differences between them, this demonstrates that being bullied is strongly related to internet-related addictions at all educational levels. Therefore, prevention programs for bullying could show a preventive effect on PIG/PSMU in all types of schools. Although specific prevention programs for internet-related addictions could address more people affected if they are carried out at lower educational levels, the high number of people affected at all educational levels shows the need to address this concern at all schools.

### Individual and moderating effects of mental health problems

Confirming previous results from other studies, we found higher odds for PIG and even more for PSMU with increasing levels of mental health problems [50, 51]. This difference between PIG and PSMU contradicts our hypothesis. However, even though this difference between the ORs was significant, it was quite small, and results were mostly overlapping. Hence, the difference between PIG and PSMU might not be of clinical relevance. Similar results were found when differentiating between internalizing and externalizing mental health problems. Since cross-sectional data were used in this study, mental health problems may either be the cause or consequence of PIG or PSMU: on the one hand, the more burdened adolescents may be, the more they might use the internet to cope with their mental health problems leading to increasingly problematic internet use. This would be in line with the I-PACE model which suggests psychopathology as a core characteristic for the development of problematic internet use [30]. Alternatively, the more addicted adolescents might be to internet applications, the more likely they might suffer from negative psychological consequences resulting from their problematic online behavior [52, 53].

Mental health problems moderated the association of victimization with both, PIG and PSMU alike with large effect sizes, confirming our hypothesis. In-depth analyses revealed that internalizing but not externalizing problems played a role in this finding. Interestingly, and in contrast to previous research, we found that victims with low mental health problems showed higher odds for problematic internet use than non-victims [54, 55]. We assume that this might be due to the finding that adolescents with more severe psychopathological difficulties generally exhibited high levels of problematic internet use – independent of their bullying experiences. One explanation based on the I-PACE model could also be that some victims successfully compensate their mental health problems by finding distraction or social connection online, as long as the gaming or social media use is relatively unproblematic [73]. However, another explanation of ours may be that adolescents who engage in more problematic online activities than their peers might be at higher risk to become a target of (cyber-)bullying due to their hobby, which might only later lead to mental health issues [80, 85]. Importantly, it should also be considered that characteristics of our sample might have contributed to these results deviating from prior studies. More research is needed to clarify the exact underlying mechanisms that may link bullying victimization, problematic internet use, and mental health problems.

### Strengths, limitations and future directions

Two major strengths of the current study are the large sample size of more than 6,500 participants, and the application of validated, widely used questionnaires. In addition, this study is one of the few comparing PIG and PSMU regarding bullying victimization in the same sample.

However, one limitation concerns the potentially reduced generalizability of our findings. Participating students attended schools which took part in the evaluation of an anti-bullying program in one German state [58]. Therefore, conclusions on adolescents in general or on other countries may be limited. This is particularly important when our results deviated from prior, more representative studies. To generalize our findings, future studies need to replicate them in randomly selected, representative samples, ideally in multiple countries.

A second limitation is that cross-sectional data was analyzed, which constitutes a barrier to causal inference. In future research, longitudinal data is needed to allow for a better understanding of the evolution and potential bidirectionality between bullying victimization and problematic internet use. In later phases of our ongoing longitudinal evaluation study, we will be able to contribute to fill this gap by analyzing individually matched data across three annual assessments, for instance, by using latent growth curves [5, 86].

Third, all variables were only assessed by self-report measures which introduces possible social desirability or recall biases. A potential risk of common methods variance was addressed by conducting Harman’s single factor tests and by using questionnaires with reverse-coded questions. Despite possible shortcomings, self-reports still appear to be the most suitable measure for sensitive topics such as bullying and psychopathological issues which are often accompanied by shame [87, 88]. In addition, deceiving others regarding one’s addictive behavior is one symptom of internet-related disorders such as IGD [19]. Nevertheless, future research may additionally conduct more objective measures to assess addictive gaming or social media use.

Fourth, our study does not account for the exact content or context of internet gaming or social media use, which might limit the interpretability of the behavioral outcomes. Future studies should consider this to gain a better understanding of potential underlying mechanisms.

Fifth, victimization was assessed by a screening questionnaire consisting of just three items, which may lack sufficient depth. However, a large number of studies used only one global item to evaluate bullying (e.g.: “How often have you been bullied?”) and the Bullying Screening [61, 62] includes a clear definition and concrete examples of bullying to ensure validity. Thus, the screening used in our study is deemed suitable for the assessment of bullying. Nonetheless, due to its primary function as a screening tool, the internal consistency was relatively low.

Finally, missing data in this study was not completely missing at random, since students in grade 5, 6 and 9 as well as B-level students had more missing data than students in grade 7 and 8 and A-level students. This could potentially bias our results. However, the proportion of missing data was very small which still justifies the analyses of the complete case sample and makes a bias unlikely [59, 60].

Beyond addressing the limitations mentioned above, future studies may compare bullying in an offline (direct, indirect bullying) versus an online context (cyberbullying) in terms of PIG and PSMU. Due to the large overlap of traditional and cyberbullying, three groups of victims may be compared: victims of only traditional bullying, only cyberbullying and those experiencing both. This, however, will result in rather complex analyses making interpretations more challenging.

Moreover, future research could consider the potential influence of further variables that have not been assessed in our study, such as the influence of low self-control [72, 89]. This may help to gain a better understanding of the interplay between personality-related variables, bullying victimization and problematic internet use.

## Conclusions

This study confirms that bullying victimization seems to be associated with both PIG and PSMU. Thus, in a clinical context, patients with internet use disorders should be questioned about their bullying experiences to better understand underlying motives. Similarly, educators may ask victims of bullying about their internet use to prevent or to uncover addictive behaviors. Generally, programs to prevent and address bullying as well as internet use disorders are needed in all age groups, regardless of educational level, gender and existing mental health problems. However, to provide targeted prevention and intervention, such programs may consider the different influences on PIG and PSMU of bullying victimization (PSMU: victimized boys; PIG and PSMU: victims with low mental health problems), gender (PIG: boys; PSMU: girls), age (PIG: younger adolescents), school type (PIG and especially PSMU: B-level schools) and mental health problems (PIG and especially PSMU: more difficulties). The school setting offers a substantial opportunity to reach a large population of youths, and to ensure a healthy and safe environment for adolescents while growing up.

## Supplementary Information

Below is the link to the electronic supplementary material.

Supplementary material 1.

## Data Availability

The datasets generated and/or analyzed during the current study are not publicly available due to ethical restrictions but are available from the corresponding author on reasonable request.

## Abbreviations

CI: Confidence interval
DSM-5: Diagnostic and Statistical Manual of Mental Disorders (5th edition)
ICC: Intraclass correlation coefficient
ICD-11: International Classification of Diseases (11th revision)
IGD: Internet Gaming Disorder
I-PACE: Interaction of Person-Affect-Cognition-Execution model
MLR: Maximum likelihood estimation with robust standard errors
OR: Odds ratio
PIG: Problematic internet gaming
PSMU: Problematic social media use
SD: Standard deviation
SDQ: Strengths and Difficulties Questionnaire
SMD: Social Media Disorder
