# Supplementary material for: Associations of bullying victimization with problematic internet gaming and problematic social media use among adolescents: moderators and differences
Source: Child Adolesc Psychiatry Ment Health. 2025 Dec 14;20:6. doi: 10.1186/s13034-025-01008-x (PMC12821830; doi:10.1186/s13034-025-01008-x)
Supplement: Supplementary file 1 — Supplementary material 1. [file 13034_2025_1008_MOESM1_ESM.docx]

**Supplementary material**

**Table A1** Questions and response format of the Bullying Screening used to classify victims of bullying

| Question # | Question and response format |
| --- | --- |
| Question 1 | The following questions refer to direct bullying. This means that others are harmed through a direct attack and that the victimized person is unable to defend him- or herself.  Some children or teenagers experience the following things repeatedly at school:  *They are threatened, blackmailed or stolen from.*  *They are insulted or called nasty names.*  *They are the victim of nasty pranks/are ridiculed.*  *They are hit, pushed around or beaten up.*  How often have these things happened to you in the last 6 months? |
|  | - Never - Rarely (1-4 times) - Sometimes (more than 4 times) - Often (2-3 times per month) - Very often (1 time per week) - Constantly (several times per week) |
| Question 2 | The following questions refer to indirect bullying. This means that the bullying is aimed at damaging a person's relationships with friends and their status within a group and the person is unable to defend him- or herself.  Some children or teenagers experience the following things repeatedly at school:  *They are deliberately excluded from meetings, parties, activities or groups.*  *They are ignored by others, others no longer want to be friends with them or do not want them in their group.*  *Nasty lies, rumors or stories are spread about them.*  How often have these things happened to you in the last 6 months? |
|  | - Never - Rarely (1-4 times) - Sometimes (more than 4 times) - Often (2-3 times per month) - Very often (1 time per week) - Constantly (several times per week) |
| Question 3 | The following questions refer to cyberbullying. This means that someone is trying to annoy or hurt another person using electronic devices (e.g., cell phones, chat messages, blogs, websites like Facebook or YouTube or emails) and the victimized person is unable to defend him- or herself.  Some children and teenagers experience the following things repeatedly at school:  *Their private emails, chat messages or text messages are forwarded to others or posted for others to see.*  *Rumors are spread about them online.*  *They receive threatening or aggressive emails, chat messages or text messages.*  *Embarrassing photos are posted online without their consent.*  How often have these things happened to you in the last 6 months? |
|  | - Never - Rarely (1-4 times) - Sometimes (more than 4 times) - Often (2-3 times per month) - Very often (1 time per week) - Constantly (several times per week) |

**Harman’s single factor test**

For the Internet Gaming Disorder Scale (IGDS)

. factor igds1 igds2 igds3 igds4 igds5 igds6 igds7 igds8 igds9, pcf

(obs=6,735)

Factor analysis/correlation Number of obs = 6,735

Method: principal-component factors Retained factors = 1

Rotation: (unrotated) Number of params = 9

--------------------------------------------------------------------------

Factor | Eigenvalue Difference Proportion Cumulative

-------------+------------------------------------------------------------

Factor1 | 3.15833 2.26663 0.3509 0.3509

Factor2 | 0.89170 0.05704 0.0991 0.4500

Factor3 | 0.83465 0.04497 0.0927 0.5427

Factor4 | 0.78968 0.03034 0.0877 0.6305

Factor5 | 0.75934 0.03233 0.0844 0.7149

Factor6 | 0.72702 0.02512 0.0808 0.7956

Factor7 | 0.70190 0.07679 0.0780 0.8736

Factor8 | 0.62511 0.11283 0.0695 0.9431

Factor9 | 0.51227 . 0.0569 1.0000

--------------------------------------------------------------------------

LR test: independent vs. saturated: chi2(36) = 9883.04 Prob>chi2 = 0.0000

Factor loadings (pattern matrix) and unique variances

---------------------------------------

Variable | Factor1 | Uniqueness

-------------+----------+--------------

igds1 | 0.6375 | 0.5936

igds2 | 0.6517 | 0.5753

igds3 | 0.6830 | 0.5335

igds4 | 0.5928 | 0.6485

igds5 | 0.5037 | 0.7463

igds6 | 0.5735 | 0.6710

igds7 | 0.5322 | 0.7168

igds8 | 0.5690 | 0.6763

igds9 | 0.5653 | 0.6804

---------------------------------------

For the Social Media Disorder Scale (SMDS)

. factor smds1 smds2 smds3 smds4 smds5 smds6 smds7 smds8 smds9, pcf

(obs=6,735)

Factor analysis/correlation Number of obs = 6,735

Method: principal-component factors Retained factors = 1

Rotation: (unrotated) Number of params = 9

--------------------------------------------------------------------------

Factor | Eigenvalue Difference Proportion Cumulative

-------------+------------------------------------------------------------

Factor1 | 3.58688 2.73613 0.3985 0.3985

Factor2 | 0.85074 0.03747 0.0945 0.4931

Factor3 | 0.81327 0.08426 0.0904 0.5834

Factor4 | 0.72901 0.01923 0.0810 0.6644

Factor5 | 0.70977 0.07930 0.0789 0.7433

Factor6 | 0.63047 0.02002 0.0701 0.8133

Factor7 | 0.61046 0.05014 0.0678 0.8812

Factor8 | 0.56032 0.05124 0.0623 0.9434

Factor9 | 0.50908 . 0.0566 1.0000

--------------------------------------------------------------------------

LR test: independent vs. saturated: chi2(36) = 1.3e+04 Prob>chi2 = 0.0000

Factor loadings (pattern matrix) and unique variances

---------------------------------------

Variable | Factor1 | Uniqueness

-------------+----------+--------------

smds1 | 0.6758 | 0.5433

smds2 | 0.6425 | 0.5872

smds3 | 0.6721 | 0.5482

smds4 | 0.5356 | 0.7131

smds5 | 0.5629 | 0.6831

smds6 | 0.6288 | 0.6046

smds7 | 0.6446 | 0.5845

smds8 | 0.6349 | 0.5970

smds9 | 0.6693 | 0.5520

---------------------------------------


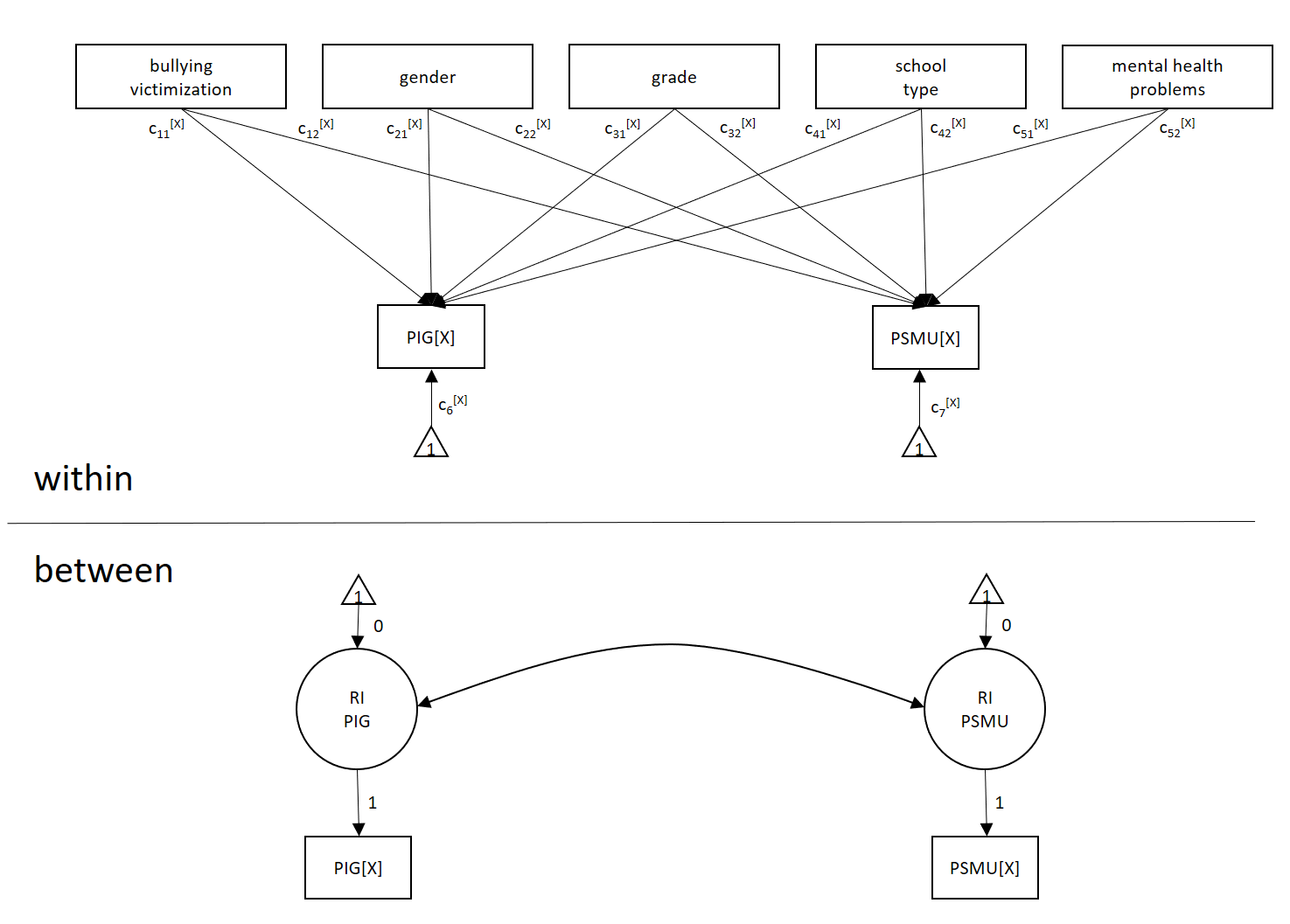


**Fig A1.** Graphical representation of the implemented model

*Note:* PIG = problematic internet gaming; PSMU = problematic social media use; RI = random intercept. Within class level (top); between class level (bottom).

PIG[X] and PSMU[X] represent the nine dichotomous PIG and PSMU items (X є {1,2,3,4,5,6,7,8,9}). Effect constraints: c_nm_^[X]^ = c_nm_^[Y]^, c_l_^[X]^ = c_l_^[Y]^, n є {1,2,3,4,5}, m є {1,2}, l є {6,7}, X,Y є {1,2,3,4,5,6,7,8,9}. For models with interactions, an additional independent variable acts on PIG[X] and PSMU[X]. For illustration purposes, grade is shown as single predictor but implemented as four dummy coded predictors.

**Table A2** Model of three bullying victimization forms and gender, grade, school type and mental health problems

| Parameter | PIG |  | PSMU |  |
| --- | --- | --- | --- | --- |
|  | *OR* (lower/ upper level) | *p* | *OR* (lower/ upper level) | *p* |
| Direct bullying | 1.08 (0.96, 1.21) | .20 | 1.04 (0.92, 1.18) | .56 |
| Indirect bullying | 1.14 (1.01, 1.30) ^b^ | .04* | 1.17 (1.03, 1.34) ^b^ | .02* |
| Cyberbullying | 1.24 (1.02, 1.50) ^b^ | .03* | 1.41 (1.14, 1.74) ^b^ | <.01** |
| Gender (male) | 2.25 (2.09, 2.43) ^c^ | <.001*** | 0.79 (0.73, 0.86) ^b^ | <.001*** |
| Grade 5 vs. 6 | 0.88 (0.79, 0.99) ^b^ | .04* | 1.01 (0.87, 1.16) | .94 |
| Grade 5 vs. 7 | 0.85 (0.76, 0.96) ^b^ | <.01** | 1.15 (1.00, 1.32) ^b^ | <.05* |
| Grade 5 vs. 8 | 0.74 (0.66, 0.83) ^b^ | <.001*** | 1.13 (0.98, 1.31) | .09 |
| Grade 5 vs. 9 | 0.65 (0.57, 0.74) ^b^ | <.001*** | 1.08 (0.94, 1.24) | .29 |
| Grade 6 vs. 7 | 0.96 (0.86, 1.07) | .49 | 1.14 (1.00, 1.30) ^b^ | <.05* |
| Grade 6 vs. 8 | 0.84 (0.75, 0.93) ^b^ | <.01** | 1.13 (0.98, 1.30) | .09 |
| Grade 6 vs. 9 | 0.73 (0.65, 0.83) ^b^ | <.001*** | 1.07 (0.94, 1.23) | .31 |
| Grade 7 vs. 8 | 0.87 (0.77, 0.98) ^b^ | .02* | 0.99 (0.86, 1.13) | .84 |
| Grade 7 vs. 9 | 0.76 (0.68, 0.86) ^b^ | <.001*** | 0.94 (0.82, 1.07) | .34 |
| Grade 8 vs. 9 | 0.88 (0.78, 1.00) ^b^ | .04* | 0.95 (0.83, 1.09) | .48 |
| School type (B-level) | 1.12 (1.04, 1.21) ^b^ | <.01** | 1.21 (1.11, 1.32) ^b^ | <.001*** |
| Mental health problems | 1.11 (1.10, 1.12) ^c^ | <.001*** | 1.13 (1.12, 1.14) ^c^ | <.001*** |
| Intercept | 0.04 (0.04, 0.05) | <.001*** | 0.03 (0.03, 0.04) | <.001*** |
| Variance random intercept |  | <.001*** |  | <.001*** |
| Covariance random intercepts |  | <.001*** |  | <.001*** |

*Note:* * *p* <.05, ** *p* <.01, *** *p* <.001. ^a^ small effect, ^b^ medium effect, ^c^ large effect. *OR* = odds ratio; PIG = problematic internet gaming; PSMU = problematic social media use. *N*=6,735

A model assuming different effects of the three bullying forms (direct, indirect, cyber) on PIG or PSMU, respectively, showed no better model fit than a model assuming same effects of all three bullying forms (χ^2^ (4) = 5.75 *p* = 0.22).

As presented in Table A2, there are significant effects for indirect as well as cyberbullying victimization for PIG and PSMU but not for direct bullying. However, comparing these effects shows no significant differences:

Regarding PIG:

- Direct versus indirect bullying: *OR* = 1.06 (0.86, 1.30), *p* = 0.58
- Direct versus cyberbullying: *OR* = 1.15 (0.90, 1.47), *p* = 0.26
- Indirect versus cyberbullying: *OR* = 1.08 (0.86, 1.37), *p* = 0.50

Regarding PSMU:

- Direct versus indirect bullying: *OR* = 1.13 (0.91, 1.39), *p* = 0.26
- Indirect versus cyberbullying: *OR* = 1.20 (0.93, 1.56), *p* = 0.17

Solely direct versus cyberbullying for PSMU revealed significant differences (*OR* = 1.36 (1.06, 1.74), *p* = 0.02, medium effect).

Furthermore, no significant differences between PIG and PSMU were found for the three bullying forms:

- Direct bullying: *OR* = 0.96 (0.85, 1.09), *p* = 0.57
- Indirect bullying: *OR* = 1.03 (0.91, 1.15), *p* = 0.67
- Cyberbullying: *OR* = 1.14 (0.93, 1.39), *p* = 0.22

**Table A3** Model of bullying victimization and gender, grade, school type, internalizing and externalizing mental health problems

| Parameter | PIG |  | PSMU |  |
| --- | --- | --- | --- | --- |
|  | *OR* (lower/ upper level) | *p* | *OR* (lower/ upper level) | *p* |
| Bullying | 1.24 (1.14, 1.36) ^b^ | <.001*** | 1.28 (1.15, 1.42) ^b^ | <.001*** |
| Gender (male) | 2.17 (2.01, 2.35) ^c^ | <.001*** | 0.75 (0.69, 0.82) ^b^ | <.001*** |
| Grade 5 vs. 6 | 0.88 (0.79, 0.99) ^b^ | .03* | 1.00 (0.87, 1.16) | 0.97 |
| Grade 5 vs. 7 | 0.85 (0.76, 0.96) ^b^ | .01** | 1.16 (1.01, 1.33) ^b^ | .04* |
| Grade 5 vs. 8 | 0.75 (0.66, 0.84) ^b^ | <.001*** | 1.15 (1.00, 1.34) | .06 |
| Grade 5 vs. 9 | 0.66 (0.58, 0.75) ^b^ | <.001*** | 1.11 (0.96, 1.28) | .16 |
| Grade 6 vs. 7 | 0.97 (0.87, 1.08) | .55 | 1.15 (1.01, 1.32) ^b^ | .03* |
| Grade 6 vs. 8 | 0.85 (0.76, 0.94) ^b^ | <.01** | 1.15 (1.00, 1.32) ^b^ | <.05* |
| Grade 6 vs. 9 | 0.75 (0.66, 0.84) ^b^ | <.001*** | 1.10 (0.96, 1.26) | .15 |
| Grade 7 vs. 8 | 0.87 (0.78, 0.98) ^b^ | .02* | 1.00 (0.87, 1.14) | .97 |
| Grade 7 vs. 9 | 0.77 (0.68, 0.87) ^b^ | <.001*** | 0.96 (0.84, 1.09) | .50 |
| Grade 8 vs. 9 | 0.88 (0.78, 1.00) ^b^ | <.05* | 0.96 (0.84, 1.10) | .56 |
| School type (B-level) | 1.12 (1.04, 1.21) ^b^ | <.01** | 1.21 (1.11, 1.32) ^b^ | <.001*** |
| Internalizing problems | 1.09 (1.08, 1.10) ^b^ | <.001*** | 1.10 (1.09, 1.11) ^b^ | <.001*** |
| Externalizing problems | 1.13 (1.12, 1.14) ^b^ | <.001*** | 1.16 (1.14, 1.17) ^c^ | <.001*** |
| Intercept | 0.04 (0.04, 0.05) | <.001*** | 0.03 (0.03, 0.04) | <.001*** |
| Variance random intercept |  | <.001*** |  | <.001*** |
| Covariance random intercepts |  | <.001*** |  | <.001*** |

*Note:* * *p* <.05, ** *p* <.01, *** *p* <.001. ^a^ small effect, ^b^ medium effect, ^c^ large effect. *OR* = odds ratio; PIG = problematic internet gaming; PSMU = problematic social media use. *N*=6,735

The model including bullying victimization, gender, grade, school type, internalizing and externalizing mental health problems showed a good model fit (χ^2^ (18) = 3043.73, *p* < .001).

Bullying victimization was significantly associated with PIG and PSMU, showing a higher risk for PIG and PSMU for victims than non-victims. These associations did not differ between PIG and PSMU (*OR* = 1.03, 95% CI: [0.93;1.13], *p* = .56).

For gender, boys had a higher risk for PIG, while girls had a higher risk for PSMU. These gender differences between PIG and PSMU were significant (*OR* = 0.35, 95% CI: [0.31;0.38], *p* < .001, large effect).

Regarding grade, a significant effect was found for PIG (χ^2^ (4) = 51.28, *p* < .001) with a tendency of lower risk for PIG at older age (see Table A3: grade 5 versus 6; grade 6 versus 7; grade 7 versus 8; grade 8 versus 9). In contrast, no significant effect was found in terms of PSMU (χ^2^ (4) = 8.36, *p* = .08). The effect of age differed significantly between PIG and PSMU (χ^2^ (4) = 95.61, *p* < .001).

For school type, B-level students had a higher risk for PIG and PSMU than A-level students. Significant differences were found between PIG and PSMU with a stronger influence of school type on PSMU than PIG (*OR* = 1.08, 95% CI: [1.00;1.17], *p* < .05, small effect).

Regarding mental health problems, the more internalizing or externalizing difficulties reported, the higher was the risk of PIG and PSMU. The associations differed significantly between PIG and PSMU with a stronger influence of internalizing or externalizing problems on PSMU than PIG (internalizing: *OR* = 1.01, 95% CI: [1.01;1.02], *p* = .04, small effect; externalizing: *OR* = 1.02, 95% CI: [1.01;1.04], *p* < .001, small effect).

**Table A4** Interaction models of bullying victimization and gender, grade, school type and mental health problems

| Parameter | PIG |  | PSMU |  |
| --- | --- | --- | --- | --- |
|  | *OR* (lower/ upper level) | *p* | *OR* (lower/ upper level) | *p* |
| Interaction bullying and gender |  |  |  |  |
| Bullying | 1.14 (1.01, 1.29) ^b^ | .04* | 1.15 (1.02, 1.31) ^b^ | .03* |
| Gender (male) | 2.18 (2.01, 2.36) ^c^ | <.001*** | 0.75 (0.69, 0.82) ^b^ | <.001*** |
| Bullying X gender (male) | 1.15 (0.98, 1.35) | .10 | 1.21 (1.01, 1.45) ^b^ | .04* |
| Grade 5 vs. 6 | 0.89 (0.79, 0.99) ^b^ | .04* | 1.01 (0.87, 1.16) | .93 |
| Grade 5 vs. 7 | 0.85 (0.76, 0.96) ^b^ | .01** | 1.15 (1.00, 1.32) ^b^ | <.05* |
| Grade 5 vs. 8 | 0.74 (0.66, 0.84) ^b^ | <.001*** | 1.14 (0.99, 1.32) | .08 |
| Grade 5 vs. 9 | 0.65 (0.57, 0.74) ^b^ | <.001*** | 1.08 (0.94, 1.25) | .26 |
| Grade 6 vs. 7 | 0.96 (0.86, 1.07) | .49 | 1.14 (1.00, 1.31) ^b^ | <.05* |
| Grade 6 vs. 8 | 0.84 (0.75 ,0.94) ^b^ | <.01** | 1.13 (0.99, 1.31) | .08 |
| Grade 6 vs. 9 | 0.74 (0.65, 0.83) ^b^ | <.001*** | 1.08 (0.94, 1.23) | .28 |
| Grade 7 vs. 8 | 0.87 (0.77, 0.98) ^b^ | .02* | 0.99 (0.87, 1.14) | .89 |
| Grade 7 vs. 9 | 0.76 (0.68, 0.86) ^b^ | <.001*** | 0.94 (0.83, 1.07) | .36 |
| Grade 8 vs. 9 | 0.88 (0.78, 0.99) ^b^ | .04* | 0.95 (0.83,1.09) | .47 |
| School type (B-level) | 1.12 (1.04, 1.21) ^b^ | <.01** | 1.22 (1.12, 1.33) ^b^ | <.001*** |
| Mental health problems | 1.11 (1.10, 1.12) ^c^ | <.001*** | 1.13 (1.12, 1.14) ^c^ | <.001*** |
| Intercept | 0.04 (0.04, 0.05) | <.001*** | 0.03 (0.03, 0.04) | <.001*** |
| Variance random intercept |  | <.001*** |  | <.001*** |
| Covariance random intercepts |  | <.001*** |  | <.001*** |
| Interaction bullying and grade |  |  |  |  |
| Bullying | 1.21 (1.01, 1.46) ^b^ | .04* | 1.41 (1.11, 1.80) ^b^ | <.01** |
| Gender (male) | 2.24 (2.08, 2.42) ^c^ | <.001*** | 0.79 (0.72, 0.85) ^b^ | <.001*** |
| Grade 5 vs. 6 | 0.86 (0.76, 0.97) ^b^ | .02* | 1.04 (0.88, 1.22) | .66 |
| Grade 5 vs. 7 | 0.84 (0.74, 0.95) ^b^ | <.01** | 1.18 (1.01, 1.38) ^b^ | .03* |
| Grade 5 vs. 8 | 0.75 (0.66, 0.86) ^b^ | <.001*** | 1.15 (0.98, 1.35) | .09 |
| Grade 5 vs. 9 | 0.66 (0.58, 0.76) ^b^ | <.001*** | 1.16 (0.99, 1.36) | .07 |
| Grade 6 vs. 7 | 0.98 (0.87, 1.10) | .68 | 1.14 (0.99, 1.31) | .07 |
| Grade 6 vs. 8 | 0.88 (0.78, 0.99) ^b^ | .04* | 1.11 (0.96, 1.29) | .18 |
| Grade 6 vs. 9 | 0.77 (0.68, 0.88) ^b^ | <.001*** | 1.12 (0.97, 1.30) | .13 |
| Grade 7 vs. 8 | 0.90 (0.80, 1.02) | .09 | 0.97 (0.85, 1.12) | .69 |
| Grade 7 vs. 9 | 0.79 (0.70, 0.90) ^b^ | <.001*** | 0.98 (0.86, 1.13) | .79 |
| Grade 8 vs. 9 | 0.88 (0.77, 1.00) | .05 | 1.01 (0.87, 1.17) | .90 |
| Bullying X grade 6 | 1.15 (0.90, 1.47) | .28 | 0.88 (0.64, 1.21) | .43 |
| Bullying X grade 7 | 1.07 (0.84, 1.36) | .60 | 0.90 (0.66, 1.22) | .49 |
| Bullying X grade 8 | 0.92 (0.70, 1.20) | .53 | 0.96 (0.70, 1.32) | .80 |
| Bullying X grade 9 | 0.91 (0.70, 1.19) | .49 | 0.73 (0.52, 1.04) | .08 |
| School type (B-level) | 1.12 (1.04, 1.21) ^b^ | <.01** | 1.21 (1.11, 1.33) ^b^ | <.001*** |
| Mental health problems | 1.11 (1.10, 1.12) ^c^ | <.001*** | 1.13 (1.12, 1.14) ^c^ | <.001*** |
| Intercept | 0.04 (0.04, 0.05) | <.001*** | 0.03 (0.03, 0.04) | <.001*** |
| Variance random intercept |  | <.001*** |  | <.001*** |
| Covariance random intercepts |  | <.001*** |  | <.001*** |
| Interaction bullying and school type | |  |  |  |
| Bullying | 1.29 (1.13, 1.47) ^b^ | <.001*** | 1.19 (1.01, 1.40) ^b^ | .04* |
| Gender (male) | 2.24 (2.08, 2.42) ^c^ | <.001*** | 0.79 (0.72, 0.86) ^b^ | <.001*** |
| Grade 5 vs. 6 | 0.88 (0.79, 0.99) ^b^ | .03* | 1.00 (0.87, 1.16) | .96 |
| Grade 5 vs. 7 | 0.85 (0.75, 0.96) ^b^ | <.01** | 1.15 (1.00, 1.32) | .05 |
| Grade 5 vs. 8 | 0.74 (0.66, 0.83) ^b^ | <.001*** | 1.14 (0.98, 1.32) | .09 |
| Grade 5 vs. 9 | 0.65 (0.57, 0.74) ^b^ | <.001*** | 1.08 (0.94, 1.25) | .28 |
| Grade 6 vs. 7 | 0.96 (0.86, 1.07) | .50 | 1.15 (1.00, 1.31) ^b^ | <.05* |
| Grade 6 vs. 8 | 0.84 (0.75, 0.93) ^b^ | <.01** | 1.13 (0.98, 1.30) | .08 |
| Grade 6 vs. 9 | 0.74 (0.66, 0.83) ^b^ | <.001*** | 1.08 (0.94, 1.23) | .28 |
| Grade 7 vs. 8 | 0.87 (0.77, 0.98) ^b^ | .02* | 0.99 (0.86, 1.13) | .87 |
| Grade 7 vs. 9 | 0.76 (0.68, 0.87) ^b^ | <.001*** | 0.94 (0.83, 1.07) | .36 |
| Grade 8 vs. 9 | 0.88 (0.78, 1.00) ^b^ | .04* | 0.95 (0.83, 1.09) | .48 |
| School type (B-level) | 1.14 (1.05, 1.23) ^b^ | <.01** | 1.19 (1.08, 1.30) ^b^ | <.001*** |
| Bullying X school type (B-level) | 0.93 (0.79, 1.10) | .39 | 1.09 (0.89, 1.33) | .42 |
| Mental health problems | 1.11 (1.10, 1.12) ^c^ | <.001*** | 1.13 (1.12, 1.14) ^c^ | <.001*** |
| Intercept | 0.04 (0.04, 0.05) | <.001*** | 0.03 (0.03, 0.04) | <.001*** |
| Variance random intercept |  | <.001*** |  | <.001*** |
| Covariance random intercepts |  | <.001*** |  | <.001*** |
| Interaction bullying and mental health problems | |  |  |  |
| Bullying | 1.71 (1.35, 2.15) ^c^ | <.001*** | 2.00 (1.47, 2.72) ^c^ | <.001*** |
| Gender (male) | 2.24 (2.07, 2.41) ^c^ | <.001*** | 0.78 (0.72, 0.85) ^b^ | <.001*** |
| Grade 5 vs. 6 | 0.88 (0.79, 0.99) ^b^ | .03* | 1.00 (0.87, 1.16) | .98 |
| Grade 5 vs. 7 | 0.85 (0.75, 0.96) ^b^ | <.01** | 1.15 (1.00, 1.32) | .05 |
| Grade 5 vs. 8 | 0.74 (0.66, 0.83) ^b^ | <.001*** | 1.13 (0.98, 1.31) | .09 |
| Grade 5 vs. 9 | 0.65 (0.57, 0.74) ^b^ | <.001*** | 1.08 (0.93, 1.24) | .31 |
| Grade 6 vs. 7 | 0.96 (0.86 ,1.07) | .49 | 1.15 (1.00, 1.31) ^b^ | .04* |
| Grade 6 vs. 8 | 0.84 (0.75, 0.93) ^b^ | <.01** | 1.13 (0.98, 1.30) | .08 |
| Grade 6 vs. 9 | 0.73 (0.65, 0.83) ^b^ | <.001*** | 1.07 (0.94, 1.23) | .30 |
| Grade 7 vs. 8 | 0.87 (0.77, 0.98) ^b^ | .02* | 0.99 (0.86, 1.13) | .86 |
| Grade 7 vs. 9 | 0.76 (0.68, 0.86) ^b^ | <.001*** | 0.94 (0.82, 1.07) | .34 |
| Grade 8 vs. 9 | 0.88 (0.78, 0.99) ^b^ | .04* | 0.95 (0.83, 1.09) | .47 |
| School type (B-level) | 1.12 (1.04, 1.21) ^b^ | <.01** | 1.21 (1.11, 1.32) ^b^ | <.001*** |
| Mental health problems | 1.11 (1.11, 1.12) ^c^ | <.001*** | 1.14 (1.13, 1.14) ^c^ | <.001*** |
| Bullying X mental health problems | 0.98 (0.97, 0.99) ^b^ | <.01** | 0.98 (0.96, 0.99) ^b^ | <.01** |
| Intercept | 0.04 (0.04, 0.05) | <.001*** | 0.03 (0.02, 0.03) | <.001*** |
| Variance random intercept |  | <.001*** |  | <.001*** |
| Covariance random intercepts |  | <.001*** |  | <.001*** |

*Note:* * *p* <.05, ** *p* <.01, *** *p* <.001. ^a^ small effect, ^b^ medium effect, ^c^ large effect. *OR* = odds ratio; PIG = problematic internet gaming; PSMU = problematic social media use. *N*=6,735

**Table A5** Interaction model of bullying victimization and internalizing mental health problems

| Parameter | PIG |  | PSMU |  |
| --- | --- | --- | --- | --- |
|  | *OR* (lower/ upper level) | *p* | *OR* (lower/ upper level) | *p* |
| Bullying | 1.61 (1.34, 1.94) ^c^ | <.001*** | 1.89 (1.47, 2.42) ^c^ | <.001*** |
| Gender (male) | 2.16 (2.00, 2.34) ^c^ | <.001*** | 0.75 (0.69, 0.81) ^b^ | <.001*** |
| Grade 5 vs. 6 | 0.88 (0.79, 0.99) ^b^ | .03* | 1.00 (0.87, 1.15) | .97 |
| Grade 5 vs. 7 | 0.85 (0.76, 0.96) ^b^ | <.01** | 1.16 (1.01, 1.33) ^b^ | .04* |
| Grade 5 vs. 8 | 0.75 (0.66, 0.84) ^b^ | <.001*** | 1.15 (1.00, 1.33) | .06 |
| Grade 5 vs. 9 | 0.66 (0.58, 0.75) ^b^ | <.001*** | 1.10 (0.95, 1.27) | .19 |
| Grade 6 vs. 7 | 0.97 (0.87, 1.08) | .55 | 1.15 (1.01, 1.32) ^b^ | .03* |
| Grade 6 vs. 8 | 0.84 (0.76, 0.94) ^b^ | <.01** | 1.15 (1.00, 1.32) | .05 |
| Grade 6 vs. 9 | 0.74 (0.66, 0.84) ^b^ | <.001*** | 1.10 (0.96, 1.26) | .18 |
| Grade 7 vs. 8 | 0.87 (0.78, 0.98) ^b^ | .02* | 0.99 (0.87, 1.14) | .93 |
| Grade 7 vs. 9 | 0.77 (0.68, 0.87) ^b^ | <.001*** | 0.95 (0.83, 1.08) | .45 |
| Grade 8 vs. 9 | 0.88 (0.78, 1.00) ^b^ | <.05* | 0.96 (0.83, 1.10) | .52 |
| School type (B-level) | 1.12 (1.04, 1.21) ^b^ | <.01** | 1.21 (1.11, 1.32) ^b^ | <.001*** |
| Internalizing problems | 1.10 (1.09, 1.11) ^b^ | <.001*** | 1.11 (1.10, 1.13) ^b^ | <.001*** |
| Bullying x internalizing problems | 0.97 (0.95, 0.99) ^b^ | <.01** | 0.96 (0.94, 0.98) ^a^ | <.01** |
| Externalizing problems | 1.13 (1.12, 1.14) ^b^ | <.001*** | 1.16 (1.14, 1.17) ^c^ | <.001*** |
| Intercept | 0.04 (0.04, 0.05) | <.001*** | 0.03 (0.03, 0.04) | <.001*** |
| Variance random intercept |  | <.001*** |  | <.001*** |
| Covariance random intercepts |  | <.001*** |  | <.001*** |

*Note:* * *p* <.05, ** *p* <.01, *** *p* <.001. ^a^ small effect, ^b^ medium effect, ^c^ large effect. *OR* = odds ratio; PIG = problematic internet gaming; PSMU = problematic social media use. *N*=6,735

The model including the interaction of bullying victimization and internalizing problems showed a significantly better fit compared to the model without the interaction (χ^2^ (2) = 12.54, *p* < .01).

A significant interaction effect was found for PIG and PSMU with stronger associations between internalizing problems and PIG or PSMU, respectively, for non-victims than victims. The interaction effects did not differ between PIG and PSMU (*OR* = 0.99, 95% CI: [0.97;1.01], *p* = .26).


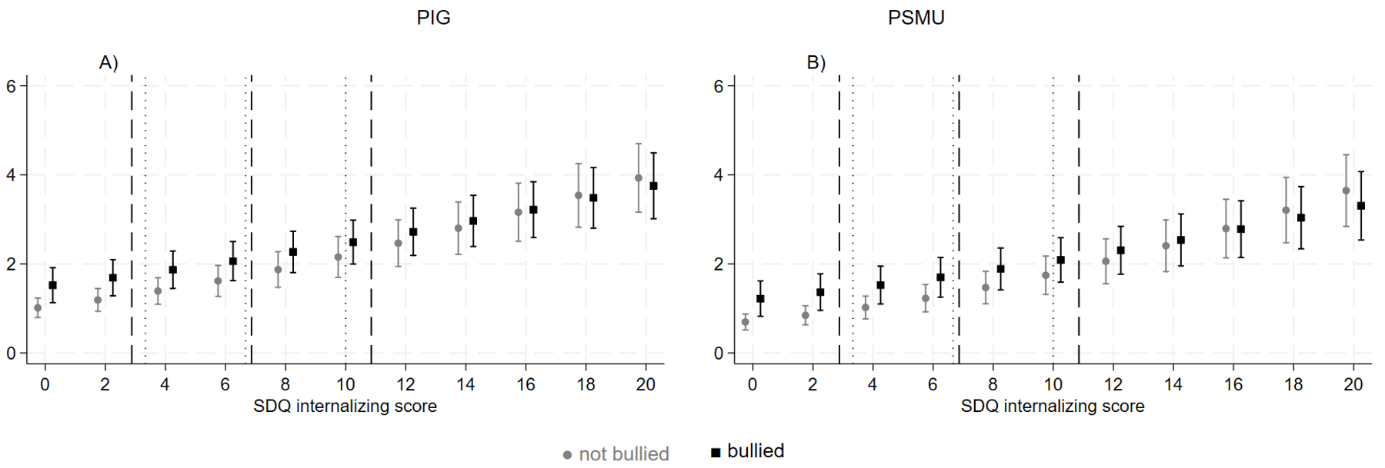


**Fig A2.** Interaction effects on problematic internet gaming and problematic social media use between bullying victimization and internalizing mental health problems

*Note:* Illustrated are marginal predicted means and 95% confidence intervals; PIG: A; PSMU: B; dashed vertical lines: mean +/- 1 standard deviation, dotted line: 25th percentile, median, 75th percentile; PIG = problematic internet gaming; PSMU = problematic social media use; SDQ = Strengths and Difficulties Questionnaire

**Table A6** Interaction model of bullying victimization and externalizing mental health problems

| Parameter | PIG |  | PSMU |  |
| --- | --- | --- | --- | --- |
|  | *OR* (lower/ upper level) | *p* | *OR* (lower/ upper level) | *p* |
| Bullying | 1.45 (1.17, 1.80) ^b^ | <.01** | 1.53 (1.19, 1.96) ^b^ | <.01** |
| Gender (male) | 2.17 (2.00, 2.35) ^c^ | <.01** | 0.75 (0.69, 0.82) ^b^ | <.001*** |
| Grade 5 vs. 6 | 0.88 (0.79, 0.99) ^b^ | .03* | 1.00 (0.87, 1.15) | .98 |
| Grade 5 vs. 7 | 0.85 (0.76, 0.96) ^b^ | .01** | 1.15 (1.01, 1.33) ^b^ | .04* |
| Grade 5 vs. 8 | 0.75 (0.66, 0.84) ^b^ | <.001*** | 1.15 (1.00, 1.33) | .06 |
| Grade 5 vs. 9 | 0.66 (0.58, 0.75) ^b^ | <.001*** | 1.11 (0.96, 1.27) | .17 |
| Grade 6 vs. 7 | 0.97 (0.87, 1.08) | .54 | 1.15 (1.01, 1.32) ^b^ | .03* |
| Grade 6 vs. 8 | 0.85 (0.76, 0.94) ^b^ | <.01** | 1.15 (1.00, 1.32) ^b^ | <.05* |
| Grade 6 vs. 9 | 0.75 (0.66, 0.84) ^b^ | <.001*** | 1.10 (0.96, 1.26) | .16 |
| Grade 7 vs. 8 | 0.88 (0.78, 0.98) ^b^ | .02* | 1.00 (0.87, 1.14) | .98 |
| Grade 7 vs. 9 | 0.77 (0.68, 0.87) ^b^ | <.001*** | 0.96 (0.84, 1.09) | .51 |
| Grade 8 vs. 9 | 0.88 (0.78, 1.00) ^b^ | <.05* | 0.96 (0.83, 1.10) | .55 |
| School type (B-level) | 1.12 (1.04, 1.21) ^b^ | <.01** | 1.21 (1.11, 1.32) ^b^ | <.001*** |
| Internalizing problems | 1.09 (1.08, 1.10) ^b^ | <.001*** | 1.10 (1.09, 1.11) ^b^ | <.001*** |
| Externalizing problems | 1.14 (1.12, 1.15) ^c^ | <.001*** | 1.16 (1.15, 1.18) ^c^ | <.001*** |
| Bullying x externalizing problems | 0.98 (0.96, 1.01) | .15 | 0.98 (0.95, 1.01) | .15 |
| Intercept | 0.04 (0.04, 0.05) | <.001*** | 0.03 (0.03, 0.04) | <.001*** |
| Variance random intercept |  | <.001*** |  | <.001*** |
| Covariance random intercepts |  | <.001*** |  | <.001*** |

*Note:* * *p* <.05, ** *p* <.01, *** *p* <.001. ^a^ small effect, ^b^ medium effect, ^c^ large effect. *OR* = odds ratio; PIG = problematic internet gaming; PSMU = problematic social media use. *N*=6,735

The model including the interaction of bullying victimization and externalizing problems showed no better fit compared to the model without the interaction (χ^2^ (2) = 2.73, *p* = .26).

No significant interaction effect was found for PIG or PSMU.


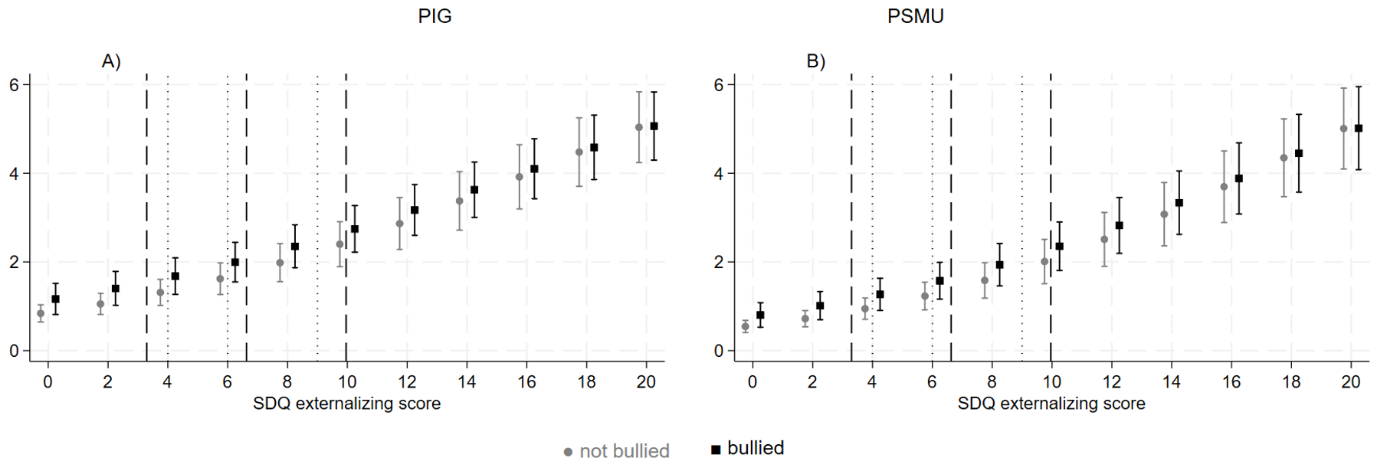


**Fig A3.** Interaction effects on problematic internet gaming and problematic social media use between bullying victimization and externalizing mental health problems

*Note:* Illustrated are marginal predicted means and 95% confidence intervals; PIG: A; PSMU: B; dashed vertical lines: mean +/- 1 standard deviation, dotted line: 25th percentile, median, 75th percentile; PIG = problematic internet gaming; PSMU = problematic social media use; SDQ = Strengths and Difficulties Questionnaire
